# Semiparametric estimation of the attributable fraction when there are interactions under monotonicity constraints

**DOI:** 10.1186/s12874-020-01118-4

**Published:** 2020-09-21

**Authors:** Wei Wang, Dylan S. Small, Michael O. Harhay

**Affiliations:** 1 Palliative and Advanced Illness Research (PAIR) Center, Perelman School of Medicine, University of Pennsylvania, Philadelphia, PA USA; 2 Department of Statistics, The Wharton School, University of Pennsylvania, Philadelphia, PA USA; 3 Department of Biostatistics, Epidemiology, and Informatics, Palliative and Advanced Illness Research (PAIR) Center, Perelman School of Medicine, University of Pennsylvania, Philadelphia, PA USA

**Keywords:** Attributable fraction, B-splines, Interaction, Monotonicity constraint, Quadratic programming

## Abstract

**Background:**

The population attributable fraction (PAF) is the fraction of disease cases in a sample that can be attributed to an exposure. Estimating the PAF often involves the estimation of the probability of having the disease given the exposure while adjusting for confounders. In many settings, the exposure can interact with confounders. Additionally, the exposure may have a monotone effect on the probability of having the disease, and this effect is not necessarily linear.

**Methods:**

We develop a semiparametric approach for estimating the probability of having the disease and, consequently, for estimating the PAF, controlling for the interaction between the exposure and a confounder. We use a tensor product of univariate B-splines to model the interaction under the monotonicity constraint. The model fitting procedure is formulated as a quadratic programming problem, and, thus, can be easily solved using standard optimization packages. We conduct simulations to compare the performance of the developed approach with the conventional B-splines approach without the monotonicity constraint, and with the logistic regression approach. To illustrate our method, we estimate the PAF of hopelessness and depression for suicidal ideation among elderly depressed patients.

**Results:**

The proposed estimator exhibited better performance than the other two approaches in the simulation settings we tried. The estimated PAF attributable to hopelessness is 67.99*%* with 95% confidence interval: 42.10*%* to 97.42*%*, and is 22.36*%* with 95% confidence interval: 12.77*%* to 56.49*%* due to depression.

**Conclusions:**

The developed approach is easy to implement and supports flexible modeling of possible non-linear relationships between a disease and an exposure of interest.

## Background

The population attributable fraction (PAF) is the fraction of disease cases in a sample that can be attributed to the exposure. The PAF is an important measure of the public health impact of an exposure on disease burden, and thus it is useful to prioritize public health interventions [1, 2]. The maximum likelihood method is commonly used to estimate the PAF. However, this approach to estimation requires a correct model for the probability of disease given the exposure and other covariates subject to the ignorable treatment assignment assumption [3] (to be reviewed in the next section). Logistic regression is typically used to model the probability of disease given the exposure and the other covariates [1, 4, 5].

The PAF of an exposure must fall between 0 and 1 by definition. A zero PAF indicates that the disease risk is irrelevant of the exposure levels. In contrast, a higher PAF value indicates a stronger association between disease risk and the exposure level. In the extreme, a PAF equal to 1 implies no disease risk when there is no exposure. If the disease risk is higher in the absence of the exposure than in the presence of the exposure, the PAF then has no proper meaning. Thus, the definition of the PAF itself also suggests a monotone relationship between the disease risk and the exposure level (a justification is presented at the end of the next section). Incorporating the monotonicity assumption into the estimation of the PAF provides performance gains when there are no other covariates [6].

In many research fields, the exposure is thought to have a monotone effect on the probability of having the disease, i.e., the probability is a monotone function of the exposure. For instance, a dose-response curve is often thought to be non-decreasing [7, 8]. One such example is the probability of suicidal ideation, which is thought to be an increasing function of both hopelessness and depression [9–12]. Hence, it is desirable to model the probability of suicidal ideation under the monotonicity constraint of both hopelessness and depression.

This situation also presents an analytic challenge. When there are other covariates, they can interact with the exposure, for instance, the interaction of two drugs [13]. In our example, hopelessness is a system of negative expectations concerning one’s future life and can cause depression. On the other hand, current depression can influence one’s hopelessness towards the future [14, 15]. Thus, an interaction between hopelessness and depression can present to have a joint effect on suicidal ideation.

These examples highlight, how in many studies, the effect of the exposure can be complicated and is not necessarily linear, including the common analysis of drug interactions [16]. We bring together several past innovations to propose a novel analytic solution. [17] study the PAF when there are joint effects or interactions. [18] develop an approach of estimating logistic regression models with interactions and monotonicity constraints. The authors [18] apply the approach to estimating the PAF and achieve substantially more accurate estimates in some settings than the usual approach which uses logistic regression without monotonicity constraints.

Semiparametric approaches have been applied to estimate relative risk functions [19], to calculate odds ratios [20], and to estimate effect measures in the presence of interactions [21]. Herein, we use B-splines [22] (see also [23, 24] and references therein), to develop a semiparametric approach to estimate the PAF in the presence of interactions with confounding. The model fitting procedure is formulated as a well studied quadratic programming problem, and, thus, can be easily solved using standard optimization packages. We implement the approach using the *R* function *solve.QP* in the package *quadprog* [25, 26]. After a simulation study, we illustrate our new method by examining hopelessness and depression for suicidal ideation among elderly depressed patients from the PROSPECT (Prevention of Suicide in Primary Care Elderly: Collaborative Trial) study [27]. Specifically, we model the interaction between hopelessness and depression under the monotonicity constraint.

## Methods

We develop the semiparametric approach to estimate PAF accounting for interactions under the monotonicity constraint using B-splines in the last two subsections. We compare the performance of the following three approaches: the approach we developed (monB), the conventional B-splines (conB) approach without the monotonicity constraint but with the same knots, and the logistic regression approach (logit). Comparisons are made through simulation studies and a case study of estimating the PAF for suicidal ideation attributable to hopelessness or depression. To save computation time, we use a small number of basis functions, quadratic B-splines with knots placed at quantiles of the distribution of unique predictor values [28, P. 24]. We use quartiles {0, 1/4, 1/2, 3/4 and 1} as the knot locations.

### Simulations

Let *Y* denote presence (1) or absence (0) of the outcome, *Z* the exposure, *X* a confounder, and *V* an additional covariate. The two interacting covariates *Z* and *X* are simulated independently from the Uniform [0,1] distribution where a 0 value is always part of the simulated *z*. The additional covariate *V* is simulated from a Bernoulli distribution with *p*=1/2. The outcome is simulated from a Bernoulli distribution with the following probability functions, A: 0.1+0.4*z*+0.3*x*−0.2*xz*+0.2*v*; B: 2(0.1+2 log(*z*/2+1))*x*/3+0.3*v*; and C: $0.8\sqrt {z+0.1}x^{2}+0.1v$. Shapes of the models at a fixed *v* are provided in Figure S1 of the supplementary material.

We examined 1000 simulations for each sample size 100 and 200. We compared the absolute value of the bias, the variance, and the mean squared error (MSE) of estimating the PAF attributable to the exposure *Z*. The true PAF values from these models are respectively 0.3, 0.4404, and 0.4616. They are calculated from (4) where the integrals are computed using *R* functions *integral* and *integral2* [29].

### Illustrative case study

In the PROSPECT study, we focus on suicidal ideation four months after the beginning of the study. We consider the 592 patients in the study with no missing data. An event was observed if the score for suicidal ideation is greater than zero [27]. Following [30], we use the Beck Hopelessness Scale [31, BHS] to measure hopeless, and the Beck Depression Inventory score [12, BDI] to measure depression. The BHS and the BDI range from 0 to 19 and from 0 to 17, respectively, where a higher value means more hopelessness or depression.

**Fig. 1:**
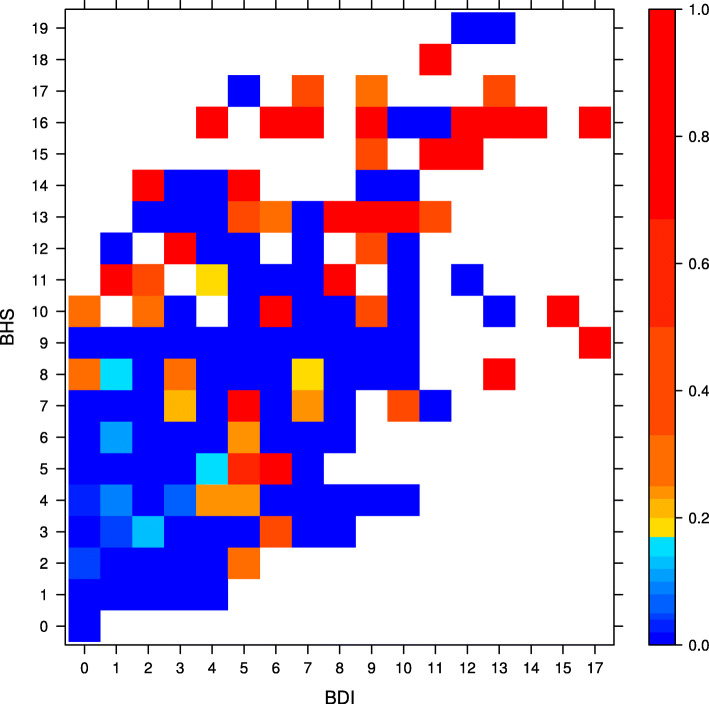
Sample average of suicidal ideation by BDI and BHS scores

**Fig. 2:**
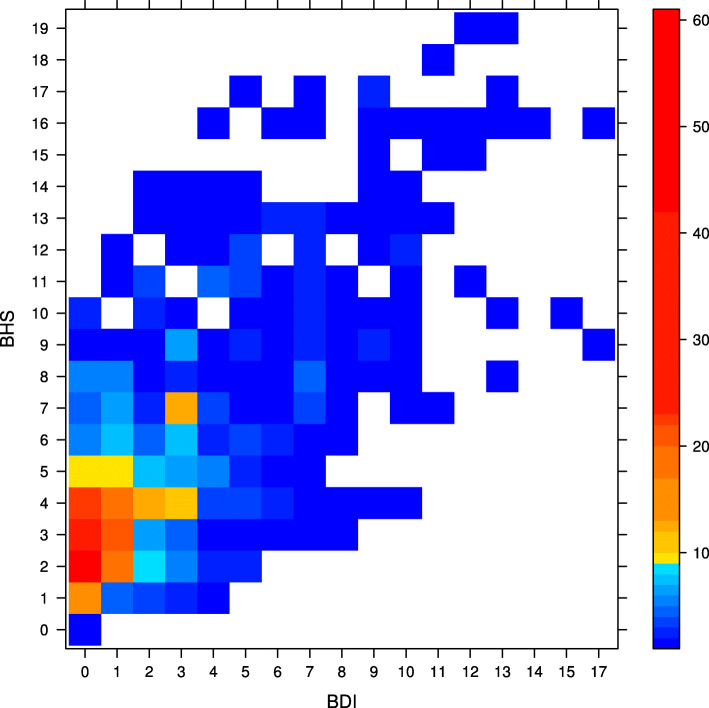
Patient frequency by BDI and BHS scores

Figure 1 shows the sample average of suicidal ideation by BDI and BHS scores, while Fig. 2 shows the corresponding patient frequency. Many patients with low BDI and BHS scores experienced no suicidal ideation. Overall, the risk of suicidal ideation increases with BDI and BHS scores, though the patient frequency decreases. The figures also show that high BHS scores are associated with high BDI scores. The PAF for hopelessness is the proportion of suicide ideation that would be prevented if all patients’ hopelessness was reduced to 0 on the BHS scale, while keeping BDI fixed. Similarly, the PAF for depression is the proportion of suicide ideation that would be prevented if all patients’ depression was reduced to 0 on the BDI scale, while BHS fixed.

### Semiparametric estimation of the probability

Suppose *Y* is distributed in the exponential family with mean *μ* [32]. The logit link function connects *μ* with the exposure and the confounder through a smooth function *f* as log[*μ*/(1−*μ*)]=*f*(*z*,*x*). [18] models *f*(*z*,*x*) parametrically assuming linearity as *f*(*z*,*x*)=*β*_0_+*β*_1_*z*+*β*_2_*x*+*β*_3_*x* *z* and estimates the coefficients under the constraint that *β*_1_*z*+*β*_3_*x* *z*≤*β*_1_*z*^∗^+*β*_3_*x* *z*^∗^ when *z*≤*z*^∗^ at every *x*. We use a flexible semiparametric approach to model a possible non-linear *f*(*z*,*x*) by linear combinations of B-splines basis functions [22] under the constraint *f*(*z*,*x*)≤*f*(*z*^∗^,*x*) when *z*≤*z*^∗^ at every *x*. Let $\boldmath {\psi }(z)=\left (\psi _{1}(z),\ldots,\psi _{P}(z)\right)'$ be a set of B-spline basis functions. Let $\boldmath {\phi }(x)=\left (\phi _{1}(x),\ldots,\phi _{Q}(x)\right)'$ be another set of B-spline basis functions. We model *f*(*z*,*x*) as

1 $$ {\begin{aligned} f(z,x)=\sum_{p=1}^{P}\sum_{q=1}^Q \psi_{p}(z)b_{p,q}\phi_{q}(x) \equiv \boldmath{\psi}(z)'\textbf{B}\boldmath{\phi}(x), \ \textbf{B}[p,q]=b_{p,q}, \end{aligned}}  $$

where **B** is the unknown coefficient matrix. Using Kronecker product ⊗ and the vectorization operator vec described in Section S1 of the supplementary material, we further obtain

$$f(z,x)=\left(\boldmath{\phi}(x)'\otimes \boldmath{\psi}(z)'\right)\text{vec} \textbf{B}\equiv\boldmath{\varphi}(z,x)'\boldmath{\beta}, $$

2 $$ \text{ where} \boldmath{\varphi}(z,x)'=\boldmath{\phi}(x)'\otimes \boldmath{\psi}(z)' \text{ and } \boldmath{\beta}=\text{vec} \textbf{B}.   $$

The maximum likelihood estimate of *β* without the constraint can be viewed as a modified iteratively weighted least squares problem [33, 34]. Derivation in Section S2 of the supplementary material shows that the constraint *f*(*z*,*x*)≤*f*(*z*^∗^,*x*) when *z*≤*z*^∗^ at every *x* can be expressed as **A***β*≥**0**, where the matrix **A** has a special pattern. The derivation in Section S3 of the supplementary material shows that the estimation procedure can be expressed as a quadratic programming problem. The approach is also capable of finding the estimate under the additional constraint that *f*(*z*,*x*) is monotone in *x*.

### Estimation of the PAF

To be general, let **X** be a vector of measured covariates. Let *Y*_0_ denote what the presence or absence would be if the exposure were to be eliminated. Let *P* be the probability measure. In particular, let *P*(*Y*_0_=1) be the “hypothetical probability of disease in the same population but with all exposure eliminated” [35, 36]. The PAF for the exposure *Z* is the proportion of disease that would be eliminated if *Z*=0,

3 $$ PAF=1-\frac{P(Y_0 = 1)}{P(Y=1)}=1-\frac{\int P(Y_0=1|\textbf{X}=\textbf{x}) d P}{P(Y=1)}.   $$

To identify the PAF based on observed data, we make the assumption that all confounders of the disease-exposure relationship are measured and contained in **X**. We assume consequently that the ignorable treatment assignment [3] holds: *P*(*Y*_0_=1|**X**=**x**)=*P*(*Y*_0_=1|**X**=**x**,*Z*=0)=*P*(*Y*=1|**X**=**x**,*Z*=0). Under this assumption, the PAF can be written as

4 $$ PAF=1-\frac{\int P(Y=1|Z=0,\textbf{X}=\textbf{x}) d P}{P(Y=1)},   $$

which is equal to the expression (2.2) for the PAF in [36]. The PAF is also written as

5 $$ {}PAF=\int \left[1-\frac{P\left(Y=1|Z=0,\textbf{X}=\textbf{x}\right)}{P\left(Y=1|Z=z, \textbf{X}=\textbf{x}\right)}\right] d P(\cdot |Y=1),   $$

where *P*(·|*Y*=1) is the conditional probability given *Y*=1 in the subpopulation of people with the disease [1, 5, 37]. We provide a proof of the equivalence between (4) and (5) in Section S4 of the supplementary material.

From a random sample of the population of size *n*, *i*=1,…,*n*, an estimate of the PAF then follows as

6 $$ {\begin{aligned} \hat{PAF}=\frac{1}{\sum_{i=1}^{n} I_{\{Y_{i}=1\}}}\sum_{\left\{ i: Y_{i}=1, 1\leq i\leq n\right\}} \left[1 -\frac{\hat{P}\left(Y_{i}=1|Z_{i}=0,\textbf{X}_{i}=\textbf{x}_{i}\right)} {\hat{P}\left(Y_{i}=1|Z_{i}=z_{i},\textbf{X}_{i}=\textbf{x}_{i}\right)}\right],  \end{aligned}}  $$

where $I_{\{Y_{i}=1\}}$ is an indicator function [18]. Justification of the convergence of the estimate (6) to the PAF in (5) is provided by [6] in the scenario of no other covariates. A similar proof adjusting for the **X** can also be derived. Thus, an accurate estimate of the probability of the disease would provide a reasonable estimate of the PAF. The defined PAF in (3) as a proportion has no practical meaning if *P*(*Y*_0_=1)>*P*(*Y*=1). Similarly in (6), occurrences of $\hat {P}\left (Y_{i}=1|Z_{i}=0,\textbf {X}_{i}=\textbf {x}_{i}\right)>\hat {P}\left (Y_{i}=1|Z_{i}=z_{i},\textbf {X}_{i}=\textbf {x}_{i}\right)$ can result in an estimated PAF out of the [0,1] range. Hence, an estimate of the probability with the monotonicity constraint ensures a reasonable estimate of the PAF.

## Results

### Simulation

Table 1 summarizes the proportion of the times that the estimated PAF was within the [0,1] range for the three approaches. As discussed, the monotonicity constraint ensures the estimated PAF is within [0,1]. The lack of a constraint results in PAF estimates that are sometimes out of the range using the logistic regression and the conventional B-splines approaches.

**Table 1:** Proportion of the times out of the 1000 simulations that the estimated PAF is between 0 and 1

		**Model**		
**Sample size**	**Approach**	**A**	**B**	**C**
n=100	logit	0.867	0.521	0.402
	conB	0.326	0.221	0.250
	monB	1	1	1
n=200	logit	0.939	0.540	0.390
	conB	0.778	0.697	0.306
	monB	1	1	1

Table 2 shows the performance of the three approaches of estimating the PAF. In the comparison, results from the conventional B-splines approach are not shown due to its large scale. Instead, we show the results from this approach by censoring the original estimate at 0 if it is negative, or at 1 if it is bigger than 1. Similarly, we obtain the censored estimate from the logistic regression approach. In general, the censored estimate improves the original estimate. Overall, the developed approach outperformed the other approaches in the settings that we examined.

**Table 2:** Comparison of the absolute value of the bias (|*Bias*|), the variance and the MSE of estimating the PAF among the logistic regression approach (logit), the conventional B-splines approach (conB), and the developed approach (monB)

		**|*Bias*|(×10**^**−1**^**)**			**Variance (×10**^**−2**^**)**			**MSE (×10**^**−2**^**)**		
**Sample size**	**Approach**	**A**	**B**	**C**	**A**	**B**	**C**	**A**	**B**	**C**
n=100	logit	1.63	3.62	8.75	10.80	37.19	94.47	13.45	50.24	170.94
	logit ^∗^	1.86	1.28	2.40	7.98	12.54	9.53	11.44	14.17	15.29
	conB ^∗^	1.74	2.84	2.64	5.18	10.09	12.86	8.20	18.17	19.80
	monB	1.48	1.53	1.65	3.69	4.94	5.25	5.88	7.28	7.96
n=200	logit	2.07	3.67	8.53	6.27	26.80	68.97	10.53	40.25	141.63
	logit ^∗^	2.14	1.74	2.85	5.35	9.79	6.96	9.93	12.81	15.09
	conB ^∗^	0.33	1.43	2.78	5.43	7.95	11.02	5.54	9.99	18.74
	monB	1.16	1.00	1.17	2.75	3.20	3.88	4.08	4.20	5.26

The logit ^∗^ and conB ^∗^ estimates are obtained by censoring the original estimates at 0 or at 1

### Case study

**Fig. 3:**
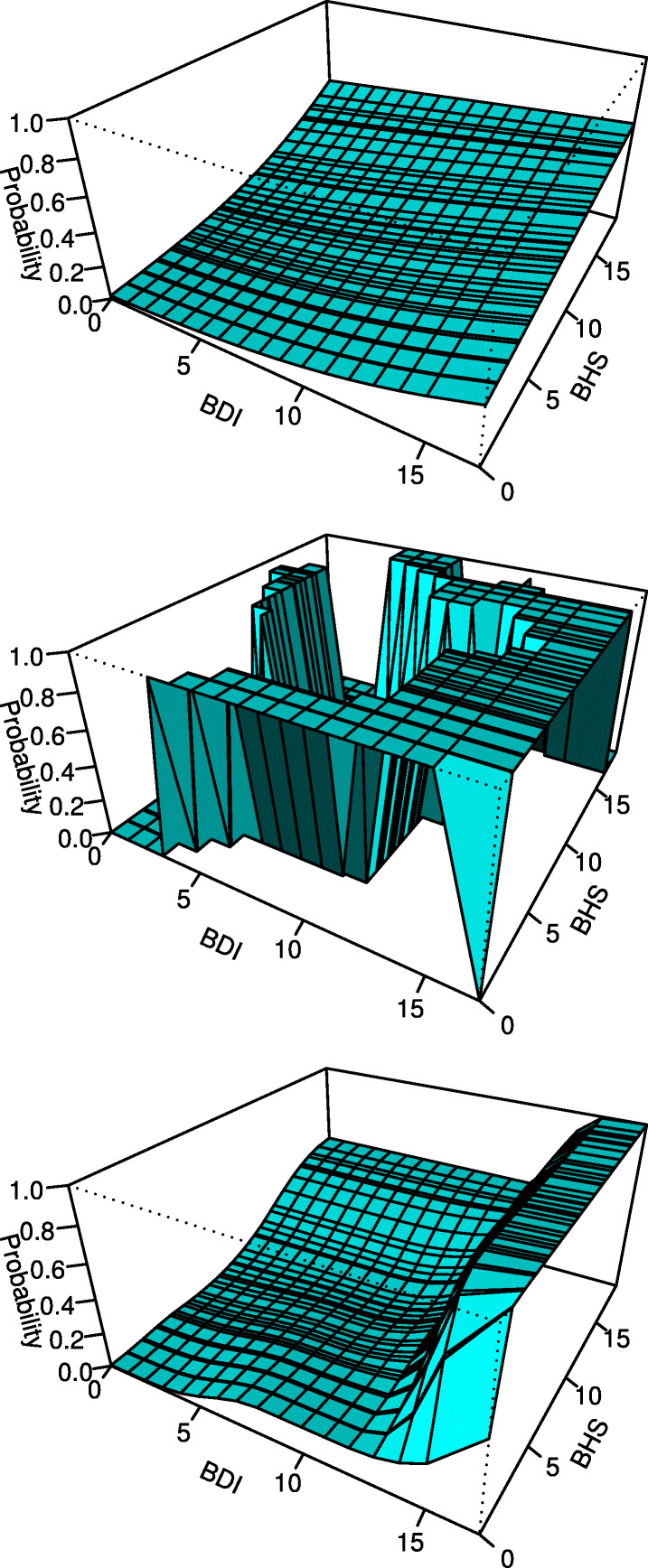
Estimated probability of suicidal ideation by the logistic regression approach (top), the conventional B-splines approach (middle), and the developed approach (bottom)

The estimated probability of suicidal ideation is shown in Fig. 3 separately by the three approaches. Both the logistic regression and the developed approach demonstrate a monotone relationship. Fitted probabilities numerically 0 or 1 occurred using the conventional B-splines approach. Table 3 summarizes the estimated PAF by the three approaches. The logistic regression produces lower PAF attributable to BHS and higher PAF attributable to BDI. Due to the numerical instability, the conventional B-splines estimated PAF is out of the [0,1] range. We examine the numerical instability in the “Discussion” section.

**Table 3:** Estimated PAF attributable to BHS and to BDI by the logistic regression approach (logit), the conventional B-splines approach (conB), and the developed approach (monB). The 95% confidence intervals are obtained from 2.5*%* and 97.5*%* quantiles of 1000 bootstrap estimates

	**Estimated PAF**	
**Estimator**	**Attributable to BHS (%)**	**Attributable to BDI (%)**
logit	63.57 (27.35, 82.20)	32.50 (-2.56, 58.13)
conB	< -100 (<-100, 100)	100 (<-100, 100)
monB	67.99 (42.10, 97.42)	22.36 (12.77, 56.49)

We use the bootstrap method to obtain the 95% confidence intervals of the estimates. The lower bound is the 2.5*%* quantile of the 1000 bootstrap estimates and the upper bound is the 97.5*%* quantile. The lower bound of the logistic regression estimated PAF attributable to BDI is negative. In fact, 28 of the estimated PAF’s are negative with the minimum being −32.45*%*. Among the 1000 estimates, two of the estimated PAF attributable to BHS are negative with the minimum being −4.27*%*.

## Discussion

We used B-splines to develop a semiparametric estimate of the probability of an event under the monotonicity constraint and accounting for interactions. The approach is solved as a quadratic programming problem and can be easily implemented using the statistical software *R*. Using a boosting technique [38, 39] implement a similar approach to estimate *β* under the same constraint **A***β*≥**0** to solve the problem defined in equation (3) of the supplementary material. In the settings that have been tried, a comparison of fitting a univariate generalized linear model under the monotonicity constraint showed that the boosting algorithm is computationally intensive [23]. A summary of other approaches in the literature for estimation when there are monotonicity constraints and interactions is provided in Section S8 of the supplementary material.

Throughout our study, we placed the knots at the quartiles of the distribution of the unique predictors. We observed similar performance of the conventional B-splines approach and the developed approach when the knots are placed at the tertiles {0, 1/3, 2/3, 1}. In the simulation study, Tables S1 and S2 of the supplementary material show that overall the developed approach has better performance. In the case study, using the developed approach, the estimated PAF attributable to BHS was 67.66*%* (43.41*%*,95.13*%*), and the estimated PAF attributable to BDI was 23.51*%* (10.92*%*,55.62*%*) (Table S3). Similar numerical instability was observed using the conventional B-splines approach (Figure S2 and Table S3). The estimated PAF is also out of the defined range between 0 and 1 using the conventional approach when knots are placed at the quantiles {0, 1/2, 1} (Table S3). Performance of the developed approach is robust to the knots placement.

## Conclusions

We developed a semiparametric estimate of the probability of an event using B-splines. Our approach can model a monotone relationship between the response and covariates, and can account for interactions. We applied the approach to estimate the PAF, and compared the performance of the estimator with the logistic regression approach and the conventional approach without the monotonicity constraint. Simulation studies showed that the developed estimator outperforms the other two approaches.

## Supplementary information

Additional file The supplementary material contains the following sections: S1, a review of the Kronecker product and the vectorization operator; S2 and S3, technical details of the developed approach; S4, proof of the equivalence between Eqs. (4) and (5); S5, a figure of the models used in the simulation studies; S6, additional simulation results where the knots are placed at the tertiles; S7, additional data analysis results where the knots are placed at the tertiles and at the quantiles {0, 1/2, 1}; S8 literature review of relevant statistical methods; and S9, *R* code to implement the developed approach.

## Data Availability

The *R* code to implement the developed approach is available in the supplementary material. The data that support the findings of this study are available from Martha Bruce, but are not publicly available. Data are however available from the authors upon reasonable request and with permission of Martha Bruce.

## Abbreviations

BDI: Beck depression inventory
BHS: Beck hopelessness scale
conB: Conventional B-splines approach without the monotonicity constraint
logit: Logistic regression
monB: Developed monotone B-splines approach
PAF: Population attributable fraction.
