# Supplementary material for: Semiparametric estimation of the attributable fraction when there are interactions under monotonicity constraints
Source: BMC Med Res Methodol. 2020 Sep 21;20:236. doi: 10.1186/s12874-020-01118-4 (PMC7507656; doi:10.1186/s12874-020-01118-4)
Supplement: Supplementary file 1 — The supplementary material contains the following sections: S1, a review of the Kronecker product and the vectorization operator; S2 and S3, technical details of the developed approach; S4, proof of the equivalence between Eqs. (4) and (5); S5, a figure of the models used in the simulation studies; S6, additional simulation results where the knots are placed at the tertiles; S7, additional data analysis results where the knots are placed at the tertiles and at the quantiles {0, 1/2, 1}; S8 literature review of relevant statistical methods; and S9, R code to implement the developed approach. [file 12874_2020_1118_MOESM1_ESM.pdf]

TECHNICAL ADVANCE

Supplementary Material of Semiparametric Estimation of the Attributable Fraction When There Are Interactions Under Monotonicity Constraints

Wei Wang[1]
, Dylan S Small[2]
and Michael O Harhay[3]

Full list of author information is available at the end of the article

S1 Kronecker Product and Vectorization Operator

The Kronecker product $\otimes$ is an operation on two matrices of arbitrary size resulting in a block matrix. Let $\mathbf{A}$ be a $m \times n$ matrix and $\mathbf{B}$ be a $p \times q$ matrix. Then the Kronecker product $\mathbf{A} \otimes \mathbf{B}$ is the $mp \times nq$ block matrix

$$\mathbf{A} \otimes \mathbf{B} = \begin{bmatrix} a_{11}\mathbf{B} & \dots & a_{1n}\mathbf{B} \\ \vdots & \dots & \vdots \\ a_{m1}\mathbf{B} & \dots & a_{mn}\mathbf{B} \end{bmatrix}.$$

For example,

$$\begin{bmatrix} 1 & 2 \\ 3 & 4 \end{bmatrix} \otimes \begin{bmatrix} 5 & 6 \\ 7 & 8 \end{bmatrix} = \begin{bmatrix} 1\times 5 & 1\times 6 & 2\times 5 & 2\times 6 \\ 1\times 7 & 1\times 8 & 2\times 7 & 2\times 8 \\ 3\times 5 & 3\times 6 & 4\times 5 & 4\times 6 \\ 3\times 7 & 3\times 8 & 4\times 7 & 4\times 8 \end{bmatrix}.$$

The vectorization operator 'vec' stacks columns of a matrix one underneath another. For instance,

$$\text{vec}\begin{bmatrix} 5 & 6 \\ 7 & 8 \end{bmatrix} = \begin{bmatrix} 5 \\ 7 \\ 6 \\ 8 \end{bmatrix}.$$

S2 Monotonicity constraint

Suppose z has an increasing effect on y at each x. Using the B-splines property [1–3], from (1) in the main article, it suffices to require an increasing order of the coefficients $\sum_{q=1}^{Q} b_{p,q}\phi_q(x)$'s as a function of p. That is, at each $p = 1, \dots, P-1$,

$$\sum_{q=1}^{Q} b_{p,q}\phi_q(x) \le \sum_{q=1}^{Q} b_{p+1,q}\phi_q(x) \Longleftrightarrow \sum_{q=1}^{Q} (b_{p,q} - b_{p+1,q})\phi_q(x) \le 0.$$

Hence to ensure monotonicity, we further require that at each p,

$$b_{p,q} - b_{p+1,q} \leq 0, \ \forall \ q = 1, \ldots, Q. \tag{1}$$

Let $\mathbf{J}$ be a $(P-1) \times P$ zero matrix except with elements -1 and 1 on the two main diagonals as

$$\mathbf{J} = \begin{pmatrix} -1 & 1 & & \\ & \ddots & \ddots & \\ & & -1 & 1 \end{pmatrix}.$$

Let $\mathbf{I}$ be an identity matrix whose dimension is indicated by its subscript. We get a $Q(P-1) \times PQ$ matrix $\mathbf{L}$ by $\mathbf{L} = \mathbf{I}_Q \otimes \mathbf{J}$. Let $\mathbf{0}$ be a column vector of 0's. The constraint (1) is then equivalent to $\mathbf{L}\boldsymbol{\beta} \geq \mathbf{0}$.

Similarly, suppose x has an increasing effect on y at each z. At each $q = 1, \ldots, Q-1$, we obtain the constraint

$$\sum_{p=1}^{P} \psi_p(z) b_{p,q} \leq \sum_{p=1}^{P} \psi_p(z) b_{p,q+1} \Longleftrightarrow \sum_{p=1}^{P} \psi_p(z)(b_{p,q} - b_{p,q+1}) \leq 0.$$

Then we require at each q that

$$b_{p,q} - b_{p,q+1} \leq 0, \forall \ p = 1, \ldots, P. \tag{2}$$

Define $\mathbf{R}$ a $P(Q-1) \times PQ$ matrix with elements -1 and 1 in certain rows and columns and element 0 elsewhere as

$$\mathbf{R} = \begin{pmatrix} -1_{1,1} & & & & 1_{1,P+1} & & & \\ & -1_{2,2} & & & & 1_{2,P+2} & & \\ & & \ddots & & & & \ddots & \\ & & & -1_{P(Q-1),P(Q-1)} & & & & 1_{P(Q-1),PQ} \end{pmatrix},$$

where the subscript represents the row and column index. Equivalence of the constraint (2) is then $\mathbf{R}\boldsymbol{\beta} \geq \mathbf{0}$. Monotonicity of y in both z and x then leads to the constraint $\begin{pmatrix} \mathbf{L} \\ \mathbf{R} \end{pmatrix} \boldsymbol{\beta} \geq \mathbf{0}$. In general, we write the constraint as $\mathbf{A}\boldsymbol{\beta} \geq \mathbf{0}$. The approach can also be applied to estimate a link function under other shape constraints by simply changing the matrix $\mathbf{A}$. For example, a diagonal matrix $\mathbf{A}$ ensures that $f(z, x) > 0$.

S3 Model fitting

Our goal is to find the maximum likelihood estimate of the unknown coefficient vector $\boldsymbol{\beta}$ under the constraint $\mathbf{A}\boldsymbol{\beta} \geq \mathbf{0}$. The procedure is similar to that in [2] when there are no confounders. Let $\Lambda(\boldsymbol{\beta})$ be the log likelihood function. We solve the optimization problem by iterations. Let the linear predictor of the i-th individual

be $\eta_i \equiv \boldsymbol{\varphi}_i'\boldsymbol{\beta}$, where $\boldsymbol{\varphi}_i = \boldsymbol{\varphi}(z_i, x_i)$. Suppose the variance of y_i is v_i. Let $w_i = (\partial\mu_i/\partial\eta_i)^2/v_i$ and $d_i = w_i(\partial\eta_i/\partial\mu_i)$.

Define $\mathbf{y} = (y_1, \ldots, y_n)'$ and $\boldsymbol{\Pi}' = (\boldsymbol{\varphi}_1, \ldots, \boldsymbol{\varphi}_n)$. We define the following quantities based on the estimate $\tilde{\boldsymbol{\beta}}$ obtained from the previous iteration, $\tilde{\boldsymbol{\mu}} = (\tilde{\mu}_1, \ldots, \tilde{\mu}_n)'$, $\tilde{\mathbf{D}} = \mathrm{diag}(\tilde{d}_1, \ldots, \tilde{d}_n)$, and $\tilde{\mathbf{W}} = \mathrm{diag}(\tilde{w}_1, \ldots, \tilde{w}_n)$. Using a Taylor expansion to the second order, maximizing the log likelihood $\Lambda(\boldsymbol{\beta})$ is then equivalent to solving

$$\min_{\boldsymbol{\beta}} \left\{ -\left[(\mathbf{y} - \tilde{\boldsymbol{\mu}})\tilde{\mathbf{D}} + \tilde{\boldsymbol{\beta}}'\boldsymbol{\Pi}'\tilde{\mathbf{W}}\right]' \boldsymbol{\Pi}\boldsymbol{\beta} + \frac{1}{2}\boldsymbol{\beta}'\boldsymbol{\Pi}'\tilde{\mathbf{W}}\boldsymbol{\Pi}\boldsymbol{\beta} \right\}. \tag{3}$$

Given $\tilde{\boldsymbol{\beta}}$ and the associated quantities, at the current iteration, the estimate of $\boldsymbol{\beta}$ is obtained by solving (3) under the constraint $\mathbf{A}\boldsymbol{\beta} \geq 0$ which is then a quadratic programming problem [4, 5].

S4 Proof

Proposition S4.1 *(5) is equivalent to (4).*

Proof:

To prove (4) and (5) are equivalent, we only need to show

$$\frac{\int P(Y=1|Z=0, \mathbf{X}=\mathbf{x})dP}{P(Y=1)} = \int \frac{P(Y=1|Z=0, \mathbf{X}=\mathbf{x})}{P(Y=1|Z=z, \mathbf{X}=\mathbf{x})} dP(\cdot|Y=1).$$

The latter can be written as

$$\frac{1}{P(Y=1)} \int I_{\{Y=1\}} \frac{P(Y=1|Z=0, \mathbf{X}=\mathbf{x})}{P(Y=1|Z=z, \mathbf{X}=\mathbf{x})} dP$$

where $I_{\{Y=1\}}$ is an indicator function. Conditioning on $(Z = z, X = x)$, the above integral becomes

$$\int E\left[I_{\{Y=1\}} \frac{P(Y=1|Z=0, \mathbf{X}=\mathbf{x})}{P(Y=1|Z=z, \mathbf{X}=\mathbf{x})} | Z=z, \mathbf{X}=\mathbf{x}\right] dP$$

which equals

$$\int P(Y=1|Z=z, \mathbf{X}=\mathbf{x}) \frac{P(Y=1|Z=0, \mathbf{X}=\mathbf{x})}{P(Y=1|Z=z, \mathbf{X}=\mathbf{x})} dP,$$

and further reduces to $\int P(Y=1|Z=0, \mathbf{X}=\mathbf{x})dP$.

S5 Simulation models

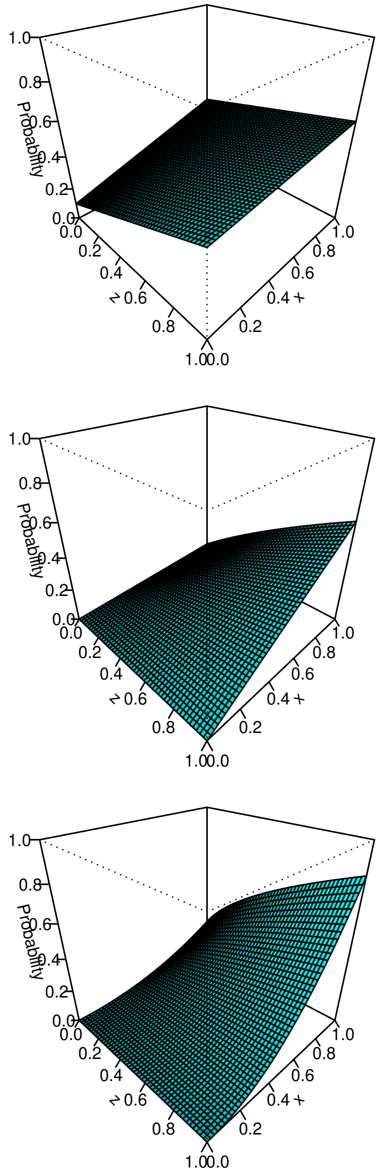

Figure S1 Models A-C (top to bottom) used in the simulations.

S6 Additional simulation results

Sample size	Approach	Model A	Model B	Model C
n=100	logit	0.867	0.521	0.402
	conB	0.732	0.661	0.390
	monB	1	1	1
n=200	logit	0.939	0.540	0.390
	conB	0.826	0.841	0.637
	monB	1	1	1

Table S1 Proportion of the times out of the 1000 simulations that the estimated PAF is between 0 and 1. The knots of the conventional B-splines approach (conB) and the developed approach (monB) are placed at the tertiles (0, $1/3$, $2/3$ and 1).

		|Bias| ($\times10^{-1}$)			Variance ($\times10^{-2}$)			MSE ($\times10^{-2}$)		
Sample size	Approach	A	B	C	A	B	C	A	B	C
n=100	logit	1.63	3.62	8.75	10.80	37.19	94.47	13.45	50.24	170.94
	logit*	1.86	1.28	2.40	7.98	12.54	9.53	11.44	14.17	15.29
	conB*	0.40	1.12	1.86	6.24	10.18	15.18	6.39	11.42	18.63
	monB	1.13	1.17	1.24	3.43	4.75	5.42	4.70	6.10	6.95
n=200	logit	2.07	3.67	8.53	6.27	26.80	68.97	10.53	40.25	141.63
	logit*	2.14	1.74	2.85	5.35	9.79	6.96	9.93	12.81	15.09
	conB*	0.24	0.85	1.37	4.77	6.92	10.75	4.82	7.63	12.62
	monB	0.89	0.72	0.81	2.52	3.09	3.91	3.31	3.61	4.56

Table S2 Comparison of the absolute value of the bias (|Bias|), the variance and the MSE of estimating the PAF among the logistic regression approach (logit), the conventional B-splines approach (conB), and the developed approach (monB). The knots of the conB and the monB approaches are placed at the tertiles (0, $1/3$, $2/3$ and 1). The logit* and conB* estimates are obtained by censoring the original estimates at 0 or at 1.

S7 Additional data analysis results

		Estimated PAF	
Knots placement	Estimator	Attributable to BHS (%)	Attributable to BDI (%)
$\{0, 1/3, 2/3, 1\}$	conB	100 (<-100, 100)	100 (<-100, 100)
	monB	67.66 (43.41, 95.13)	23.51 (10.92, 55.62)
$\{0, 1/2, 1\}$	conB	18.13 (-130.99, 86.01)	-17.20 (-95.91, 56.42)
	monB	64.64 (35.51, 90.92)	25.20 (9.48, 53.73)

Table S3 Estimated PAF attributable to BHS and to BDI by the conventional B-splines approach (conB) and the developed approach (monB). The knots are placed at the quantiles. The 95% confidence intervals are obtained from 2.5% and 97.5% quantiles of 1000 bootstrap estimates.

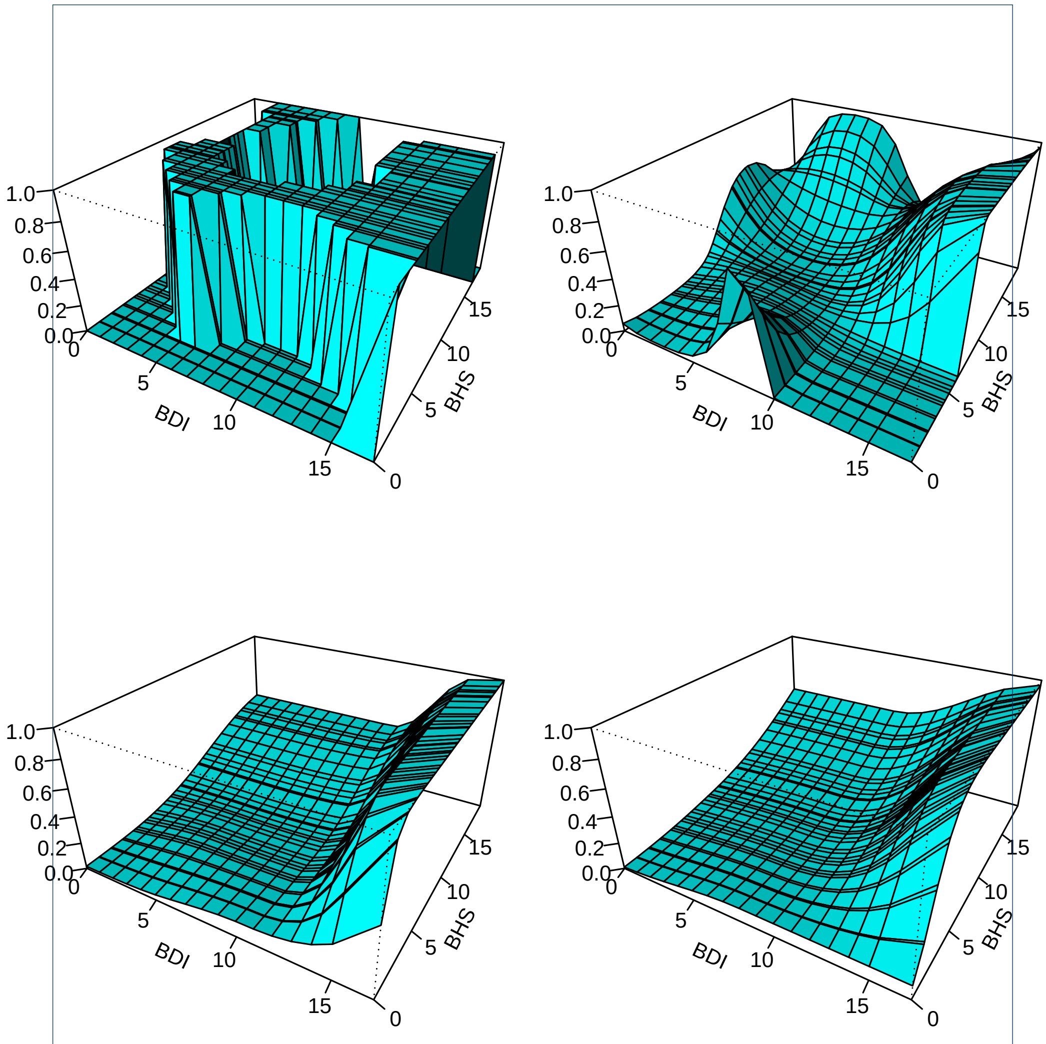

Figure S2 Estimated probability of suicidal ideation by the the conventional B-splines approach (top), and the developed approach (bottom). The knots are placed at the quantiles: $\{0,\ 1/3,\ 2/3,\ 1\}$ (left), and $\{0,\ 1/2,\ 1\}$ (right).

S8 Literature Review

Modeling the interaction between two covariates subject to the monotonicity constraint is of increasing research interest. [6] use tensor product I-splines to model the interaction between cache architecture parameters such as cache size and the number of CPUs. Monotonicity is guaranteed if the estimated regression coefficients are non-negative. In this approach, the response depends monotonically on both covariates simultaneously. The approach lacks the flexibility when only one covariate is monotonic or both covariates are monotonic but in a reverse direction. [7] use tensor product P-splines in the regression model where extra asymmetric discrete penalty terms are introduced. Associated with the penalty term is a user defined parameter which has to be tuned to see if the monotonicity constraint is violated.

In the approach of [8], an initial unconstrained smoothing estimate is obtained first. Then one dimensional monotonization procedure using kernel densities is applied successively to each of the monotonic covariates. The order of which covariate is monotonized first may have an influence on the final estimate, and in that case an averaged estimate could be used.

[3] propose a monotone smoothing method based on P-splines focusing on additive models. In the spline basis expansion, the original coefficients (starting from the second) are transformed by the exponential function. The approach defines a new fitting algorithm and facilitates efficient estimation of the associated smoothing parameters. An observation from the authors is that sometimes the algorithm can cause computational instability such as non-convergence of Fisher scoring and indefiniteness of the Hessian matrix of the log-likelihood. The method is implemented in the package *scam* of the statistical software *R*. [9] uses "warped-plane spline" with a cone projection algorithm implemented in the "cgam" package of *R*.

S9 R code

```
library(quadprog)
library(splines)
library(Matrix)
library(magic)
library(MASS)
lAm=function(p,q){
sma=matrix(0,p-1,p)  # p>1
for(i in 1:(p-1)){
sma[i,i]=-1
sma[i,i+1]=1
}
ma=kronecker(diag(1,q),sma)
return(ma) }
rAm=function(p,q,pq){
pq1=p*(q-1)
ma=matrix(0,pq1,pq)  # q>1
for(j in 1:pq1){
  ma[j,j]=-1
  ma[j,j+p]=1 }
return(ma) }
bAm=function(p,q,pq){
rbind(lAm(p,q),rAm(p,q,pq)) }
ocf=function(x,K){
ux=sort(unique(x))
oknots=quantile(ux,seq(0,1,length=K+2))
iknots=oknots[-c(1,K+2)]
nor=2
di=K+nor+1
ba=bs(x,knots=iknots,degree=nor,intercept=T)
if(0%in%x){
```

```
ba0=predict(ba,0)
} else {
ba0=predict(ba,ux[1])}
beval=predict(ba,ux)
return(list(ba=ba,di=di,beval=beval,ba0=ba0)) }
bcf=function(lx,rx,lK,rK,n){
lo=ocf(lx,lK)
lba=lo$ba
lbe=lo$beval
lba0=c(lo$ba0)
ro=ocf(rx,rK)
rba=ro$ba
rbe=ro$beval
p=lo$di
q=ro$di
pq=p*q
lrp=lrp0=matrix(0,n,pq)
  for(i in 1:n){
   lrp[i,]=kronecker(c(rba[i,]),c(lba[i,]))
   lrp0[i,]=kronecker(rba[i,],lba0)}
   #lrr0[i,]=kronecker(rba0,lba[i,]) }
return(list(lrp=lrp,lrp0=lrp0,lbe=lbe,rbe=rbe,p=p,q=q,pq=pq)) }
xwxfun=function(lrp,beta.vec,lbeta,n){
thr=1e-10
pi.vec=c(logit.fun(lrp%*%beta.vec))
wi.vec=c(pi.vec*(1-pi.vec))
cp=(abs(pi.vec-1)<thr)
cn=(abs(pi.vec)<thr)
if(any(cp)){    pi.vec[cp]=1
                wi.vec[cp]=thr}
if(any(cn)){    pi.vec[cn]=0
                wi.vec[cn]=thr}
pm=array(0, c(lbeta,lbeta,n))
for(i in 1:n){
bb=lrp[i,]
pm[,,i]=outer(bb,bb,"*")*wi.vec[i] }
xwx=apply(pm,c(1,2),sum)
xwx=(xwx+t(xwx))/2
if(min(eigen(xwx)$values)<1e-7) xwx=as.matrix(nearPD(xwx)$mat)
return(list(xwx=xwx,pi.vec=pi.vec,wi.vec=wi.vec)) }
dmv=function(lrp,y,beta.vec,lbeta,n){
xo=xwxfun(lrp,beta.vec,lbeta,n)
xwx=xo$xwx
pi.vec=xo$pi.vec
dm=xwx
dv=c(xwx%*%beta.vec)+(y-pi.vec)%*%lrp
```

```
return(list(dm=dm,dv=c(dv),pi.vec=pi.vec)) }
ufun=function(lrp,y,n,itn,eps){
lbeta=ncol(lrp)
beta.vec=rep(0,lbeta)
dif.beta=rep(1,lbeta)
nuit=0
while((nuit<itn)&(any(dif.beta>eps))){
dob=dmv(lrp,y,beta.vec,lbeta,n)
sov=solve(dob$dm,dob$dv)
dif.beta=abs(beta.vec-sov)
beta.vec=sov
nuit=nuit+1 }
return(list(dif.beta=dif.beta,beta.vec=beta.vec,
            pi.vec=dob$pi.vec,nit=nuit)) }
mfun=function(lrp,y,n,Cm,itn,eps){
lbeta=ncol(lrp)
beta.vec=rep(0,lbeta)
dif.beta=rep(1,lbeta)
nuit=0
while((nuit<itn)&(any(dif.beta>eps))){
dob=dmv(lrp,y,beta.vec,lbeta,n)
sov=solve.QP(Dmat=dob$dm,dvec=dob$dv,Amat=t(Cm))
dif.beta=abs(beta.vec-sov$solu)
beta.vec=sov$solu
nuit=nuit+1 }
return(list(dif.beta=dif.beta,beta.vec=beta.vec,
            pi.vec=dob$pi.vec,nit=nuit))  }
Bsf=function(bt,p,q,lub,rub){
B=matrix(bt,p,q)
bsf=lub%*%B%*%t(rub)
return(bsf)
 }
logit.fun=function(x.c){1/(1+exp(-x.c))}
eaf.fun=function(yi,prob.est,prob.est0){
indy=which(yi==1)
prob.est=prob.est+(1e-10)*(prob.est==0)
denom=1-prob.est0/prob.est
mean(denom[indy])
}
spafest=function(lx,rx,y,lK,rK,n,xv=NULL){
itn=100
eps=1e-7
bo=bcf(lx,rx,lK,rK,n)
lrp=bo$lrp
lrp0=bo$lrp0
p=bo$p
```

```
q=bo$q
pq=bo$pq
Cm=bAm(p,q,pq)
if(!is.null(xv)){
ap=ncol(data.matrix(xv))
Cm=cbind(Cm,matrix(0,ncol=ap,nrow=nrow(Cm)))
lrp=cbind(lrp,xv)
lrp0=cbind(lrp0,xv)
}
elrp=svd(lrp)$d
chd=(max(elrp)/min(elrp))^2
uo=ufun(lrp,y,n,itn,eps)
uest=uo$beta.vec
upi=uo$pi.vec
ul0=logit.fun(lrp0%*%uest)
uf=eaf.fun(y,upi,ul0)
mo=mfun(lrp,y,n,Cm,itn,eps)
mest=mo$beta.vec
mp=mo$pi.vec
ml0=logit.fun(lrp0%*%mest)
mf=eaf.fun(y,mp,ml0)
return(list(est=c(uf,mf),chd=chd))}
```

Author details
[1]Palliative and Advanced Illness Research (PAIR) Center
Perelman School of Medicine, University of Pennsylvania, Philadelphia, PA, USA. [2]Department of Statistics, The Wharton School, University of Pennsylvania, Philadelphia, PA, USA. [3]Department of Biostatistics, Epidemiology and Informatics, Palliative and Advanced Illness Research (PAIR) Center, Perelman School of Medicine, University of Pennsylvania, Philadelphia, PA, USA.

References

1. de Boor, C.: A Practical Guide to Splines. Springer, New York (1978)
2. Wang, W., Small, D.: Monotone B-spline smoothing for a generalized linear model response. The American Statistician **69**(1), 28–33 (2015)
3. Pya, N., Wood, S.N.: Shape constrained additive models. Statistics and Computing **25**(3), 543–559 (2015)
4. Goldfarb, D., Idnani, A.: Dual and primal-dual methods for solving strictly convex quadratic programs. In: Hennart, J.P. (ed.) Numerical Analysis, pp. 226–239. Springer, Berlin (1982)
5. Goldfarb, D., Idnani, A.: A numerically stable dual method for solving strictly convex quadratic programs. Mathematical Programming **27**, 1–33 (1983)
6. Gluhovsky, I.: Smooth isotonic additive interaction models with application to computer system architecture design. Technometrics **48**(2), 17–192 (2006)
7. Bollaerts, K., Eilers, P.H.C., Mechelen, I.V.: Simple and multiple p-splines regression with shape constraints. British Journal of Mathematical and Statistical Psychology **59**, 451–469 (2006)
8. Dette, H., Scheder, R.: Strictly monotone and smooth nonparametric regression for two or more variables. Canadian Journal of Statistics **34**(4), 535–561 (2006)
9. Liao, X., Meyer, M.: cgam: An R package for the constrained generalized additive model. Journal of Statistical Software, Articles **89**(5), 1–24 (2019)
